# The impact of climate change on food security in South Africa: Current realities and challenges ahead

**DOI:** 10.4102/jamba.v9i1.411

**Published:** 2017-08-11

**Authors:** Tshepo S. Masipa

**Affiliations:** 1 Department of Economics, University of Limpopo, South Africa

## Abstract

This article aims to examine the impact of climate change on food security in South Africa. For this purpose, the article adopted a desktop study approach. Previous studies, reports, surveys and policies on climate change and food (in)security. From this paper’s analysis, climate change presents a high risk to food security in sub-Saharan countries from crop production to food distribution and consumption. In light of this, it is found that climate change, particularly global warming, affects food security through food availability, accessibility, utilisation and affordability. To mitigate these risks, there is a need for an integrated policy approach to protect the arable land against global warming. The argument advanced in this article is that South Africa’s ability to adapt and protect its food items depends on the understanding of risks and the vulnerability of various food items to climate change. However, this poses a challenge in developing countries, including South Africa, because such countries have weak institutions and limited access to technology. Another concern is a wide gap between the cost of adapting and the necessary financial support from the government. There is also a need to invest in technologies that will resist risks on food systems.

## Introduction

The issues of climate change and its impact on food security are increasingly recognised in different parts of the world, including Africa. Africa is epitomised as the most vulnerable continent to climate changes (Bwalya 2013). Other studies also project that Africa is highly vulnerable to climate changes (Intergovernmental Panel on Climate Change [IPCC] 2007; Liliana 2005; Vogel 2005; World Bank 2016). The studies mainly found that climate changes have a severe impact on agricultural land, which ultimately affects food security. The IPCC (2007) reports that in sub-Saharan Africa, agricultural productivity will decline from 21% to 9% by 2080. Liliana (2005) indicates that about two-thirds of Africa’s arable land is expected to be lost by 2025 because of the lack of rainfall and drought. South Africa is not immune to these impacts. It is worth noting that global population is continuing to rise and the rates at which climate change and production of agriculture and food have been increasing are not equal. The World Food Programme (WFP 2016) report shows that crop production yield per hectare is, on average, increasing at a rate below that of global populations, implying that food production, which has been unable to meet global demand, will struggle to do so in the immediate future, leaving millions of people and numerous countries facing the stark reality of having reduced food security. Against this backdrop, the article seeks to examine the impact of climate change on food security in South Africa. The need to conduct the current article is further accentuated by the recent occurrence of drought and the lack of rainfall that affected South Africa in 2016.

Apart from the ‘Introduction’ section, the remainder of the article is structured as follows: The ‘Contextualisation of food security, accessibility, availability and utilisation’ section conceptualises the term food security, in terms of food accessibility, availability and utilisation. The ‘Climate change and food security: Sub-Saharan Africa’s experiences’ section presents the impacts of climate changes on agriculture and food security in various African countries. The ‘Status of food (in)security in South Africa’ section reviews the status of food security in South Africa. ‘The role of Department of Agriculture, Forestry & Fisheries’ section outlines the role of the South African government, particularly the Department of Agriculture, Forestry & Fisheries (DAFF) to address food (in)security; and lastly the ‘Conclusion and recommendations’ section provides the conclusion and recommendations.

## Contextualisation of food security, accessibility, availability and utilisation

The term food security was established in the 1960s in the international development literature (Osman 2002, cited in Le Page 2004). According to the World Food Summit (1996), food security exists when all people, at all times have access to sufficient nutritious food to meet their dietary needs and food references for an active and healthy life. Schmidhuber and Tubiello (2007) add to the concept of food security by arguing it is affected by a multitude of factors which include the ability to be self-sufficient in food production through own production, accessibility to markets and the ability to purchase food items. Anderson (1990) defines food security in terms of the ability of individuals to obtain sufficient food on a day to day basis.

It is worth pointing out that food availability is often used to measure food security; hence the concepts are often used interchangeably. Thompson, Berrange and Ford (2010) clearly distinguish between the two by defining food availability in terms of the existence of sufficient quantities of food with appropriate quality. Similarly, the Human Sciences Research Council (HSRC) defines food security in terms of three dimensions, namely food availability, food access and food use. The council suggests that a country must have sufficient quantities of food available on a consistent basis at both national and household level. Food access, on the other hand, refers to the ability of the nation and its households to acquire sufficient food on a sustainable basis (Aliber 2010). According to Ludi (2009), access to food refers to the ability of the country, communities and individuals to purchase food in sufficient quantities and quality. Another concept of food security involves food utilisation, which Negin et al. (2009) argue depends on how food is used, noting that although food availability and accessibility are necessary conditions for food utilisation, they are not sufficient conditions to reduce malnutrition. Wlokas (2008), on the other hand, argues that the direct impact of climate change on food security is through food availability due to changes in agricultural productivity. Similarly, the Food and Agriculture Organization (FAO 2011) reported that climate change affects the production rate and patterns of different food items.

Smith, Pointing and Maxwell (1993) define food security within the context of national food self-reliance, suggesting that a country should be able to produce and distribute adequate food that is needed by all its citizens. Reddy and Moetsane (2009), on the contrary, argue that food security does however not guarantee food security at a household level. The United Nation Development Programme (UNDP 2006) indicates that the concept of food security is closely linked to poverty, noting that the two concepts are interrelated and have an influence on one another. The report further reveals that poverty and unemployment have strong relationship with food insecurity, indicating that it begins with the loss of employment, which, in turn, leads to a significant degradation in the living standard.

From the preceding concepts, food security can briefly be defined in terms of food accessibility, availability, stability, utilisation and affordability. This is succinctly summarised by Ziervogel (2009), noting that food security is not just about food availability (production, distribution and exchange) but also about access (affordability, allocation and preference) and utilisation (food safety, nutrition and social value). In addition, the World Bank (2016) reported that climate change affects food utilisation capacity through changes in production rate and the pattern of various food items, and this, in turn, affects the nutritional requirements of the population.

## Climate change and food security: Sub-Saharan Africa’s experiences

The United Nations Development Report reported that one in four households in sub-Saharan Africa cannot access adequate food (United Nations 2012). The IPCC (2007) shows that Southern Africa has higher climate change vulnerability and predicts that the consequences could be severe, exerting far-reaching impacts on the livelihoods of many people. According to the IPCC (2007), agricultural productivity will decline from 21% to 9% by 2080 due to climate change in sub-Saharan Africa. The report indicates that rising temperatures in precipitation are likely to reduce the production of stable food by up to 50%. In Tanzania, Pedram, David and Navin (2011) reported that by 2050, the projected increase of 2 °C will reduce the average production of maize, sorghum and rice by 13%, 8.8% and 7.6%, respectively. Knueppel, Demment and Kaisser (2009) argue that lower levels of education attainment are directly linked with the high food insecurity in Tanzania.

A study by Gutu, Bezabih and Mengistu (2012) reveals that in Ethiopia, food production faces severe challenges due to climate change, noting that the annual production losses to climate variability significantly increase from year to year. The European Commission (2009) report similarly shows that climate change in Africa will reduce crop yields and in turn will increase the price of food that will force people to change production and consumption patterns. Vogel (2005) argues that sub-Saharan Africa is highly vulnerable to food insecurity, pointing out that drought, flooding and pest outbreaks are some of the stressors on food security. Schmidhuber and Tubiello (2007) argue that global warming will have significant negative effects on food security, with an estimate of between 5 and 170 million people at risk of hunger by the year 2080. In South Africa, Hendriks (2005) argues that South Africa is nationally food secure; however, between 58% and 73% of households experience food insecurity.

From the preceding, it is clear that climate change presents a high risk on food security in sub-Saharan countries, from crop production to food distribution and consumption. To mitigate these risks, there is therefore a need for an integrated policy approach to protect the arable land against global warming. In addition, rural small-scale farmers need to be given more support to improve their agricultural production, particularly at household level.

**FIGURE 1:**
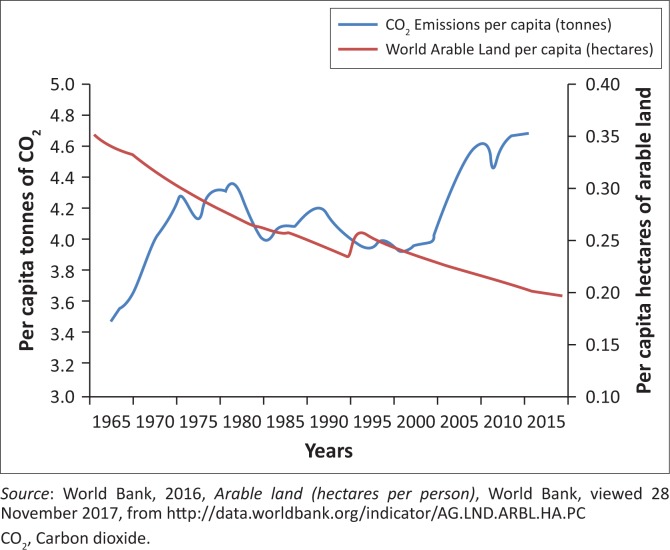
Carbon dioxide emissions and arable land.

From Figure 1, it can be denoted that since 1965, Carbon dioxide (CO_2_) emissions have been considerably low and the amount of arable land was high. However, since 2001, CO_2_ emissions have been volatile and have increased, and the amount of arable land has decreased significantly. This decrease is caused by an increase in global warming (World Bank 2016). Drought severity has increased with global warming, degrading available arable land. Many households in rural areas are dependent largely on subsistence farming for survival, and with a decrease in arable land, these households will face a lack of food security.

**FIGURE 2:**
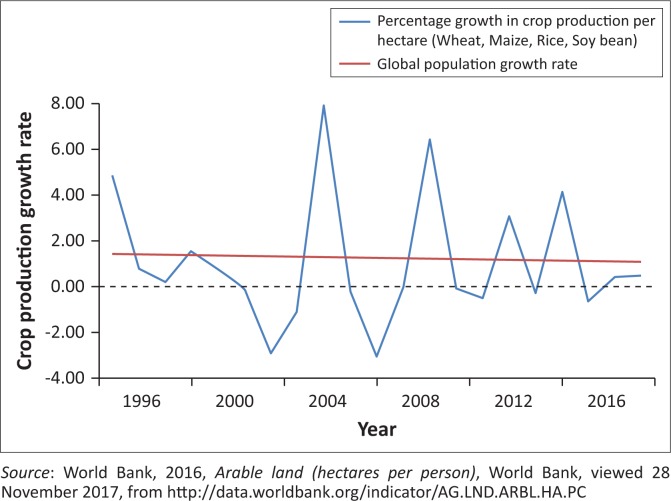
Crop production and population growth.

Global warming will have significant negative effects on food security, with estimates of an additional amount of between 5 and 170 million people at risk of hunger by the year 2080 (Statistical Review on World Energy 2016). To mitigate the impact of CO_2_ emissions on food security, Barrett (2002) suggests that there should an implementation of a policy targeting the reduction of greenhouse gas emissions, as well as maintenance and recovery of soil in areas affected by climate change. This would assist subsistence farmers which are in need of increased food production to be a more appropriate solution. Figure 2 illustrates trends of crop production and population growth.

Figure 2 illustrates that crop production per hectare has historically been volatile, while population growth rate has been relatively constant over the past 20 years. From Figure 1, it can be denoted that over the last two decades, the population has, on an average, been growing at a rate higher than that of crop production. The production of crops comprises maize, rice, wheat and soybean. The challenge of food security is exacerbated by an increase in population which is greater than the rate of food production, resulting in an increase in the number of households to feed. In essence, the demand for food exceeds the supply of food, thus leading to food shortage. This is confirmed by the World Bank (2016), reporting that global population continues to rise; however, the growth rate between food production and population growth is not equal. The crop production yield per hectare is, on average, increasing at a rate below that of global population.

Table 1 shows that over the past two decades, the population has on average been growing at a rate higher than that of crop yields. From 1996 to 2016, the average growth of crop production is 1.05% and of population is 1.27%. This raises a concern as it implies that food security is seriously challenged, as the population growth is greater than the crop production. This implies that food production will not be able to meet global demand for food, leaving millions of people having reduced food security.

**TABLE 1:** Crop yield growth rate per hectare and population growth rate.

Average	Crop yield growth rate per hectare	Population growth rate
1996–2016 Average	1.05%	1.27%

*Source*: World Bank, 2016, *Arable land (hectares per person)*, World Bank, viewed 28 November 2017, from http://data.worldbank.org/indicator/AG.LND.ARBL.HA.PC and Organisation for Economic Co-operation and Development (OECD), 2016, *Crop production*, viewed 06 June 2017, from https://data.oecd.org/agroutput/crop-production.htm

Lobell et al. (2008) argue that to increase food production, yield per hectare must increase. If yield per hectare increases at a rate slower than that of population, then food shortages experienced throughout the world will only get worse, aided by global warming. The effects of global warming on crops depend greatly on region and type of crop. However, it seems that poorer countries, more prone to be affected by global warming, need to adjust their crops to a greater extent than their more developed counterparts (Lobell et al. 2008).

To address some of the aforementioned challenges, the Department of Environmental Affairs (2013) reported that farmers have adopted various strategies to mitigate the impact of climate change on their farming, which include:

- reducing planting areas of certain crops, such as wheat
- planting crop varieties with a shorter growing period
- delaying the start of planting according to rainfall
- investing in additional machinery to shorten planting time
- collecting rainwater by creating furrows near planted areas
- increasing the use of irrigation.

## Status of food (in)security in South Africa

The South African government committed itself to half poverty levels between 2004 and 2014. In his State of the Nation Address (2014), the president of the Republic of South Africa, Jacob Zuma, noted that food security was re-prioritised as one of the top priorities for the government. This is because climate change and its impact on food in(security) is increasingly recognised as a major concern in different parts of the world, including South Africa. One of the main components in meeting that goal is household food security. Compared to other African countries, South Africa is largely considered as a food secure nation, producing sufficient staple foods and having the capacity to import food in order to meet the basic nutritional requirements of its population (FAO 2008). Hart and Aliber (2009) similarly argue that South Africa seems to be food secured at the national level, but the same cannot be said at household level, particularly in rural areas, where majority of the people largely depend on agriculture. Landman (2004), however, found that food security is a serious challenge that still persists in South Africa.

In addition, embedded in Section 26 and 27 1(b) of the Constitution of the Republic of South Africa of 1996 is the right to access sufficient food and water. This is also in line with South Africa’s millennium development goals of reducing poverty by half by 2015. The national food security indicators reported that South Africa has been able to meet the food needs of its growing population over the years, there are however no clear statistics to ascertain food security at household level (Statistics South Africa 2009). Hendriks (2005) found that in South Africa, a large proportion of households in rural areas are vulnerable to incidences of food insecurity.

Knueppel et al. (2009) and De Cock (2012) similarly found that South Africans living in rural households are most strongly affected by climate change. More worryingly, Earl (2011) noted that hunger and malnutrition are still prevalent in South Africa and this is as a result of inequalities in accessing productive land.

**FIGURE 3:**
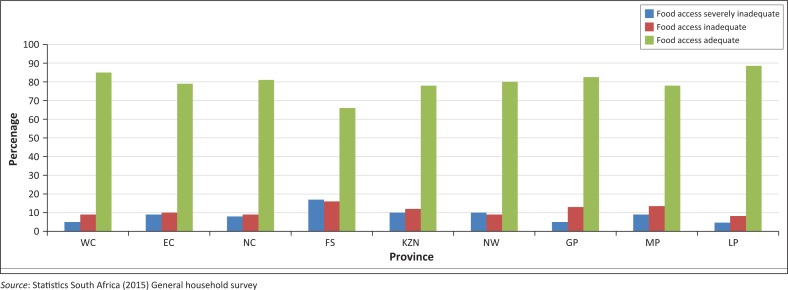
Household food adequacy by province over the period 2009–2015.

Demetre, Yul and Zandile (2009) ascertained that more than 14 million people, or about 35% of the South African population, are vulnerable to food insecurity. According to the authors, an individual is classified to be food insecure if he or she receives less than 2261 Kilocalories per day, which is equivalent to R211 per person per month. In Johannesburg, Rudolph (2012) revealed that there is a strong relationship between employment, income and food insecurity. His study concluded that members of households with full time jobs were more likely to be food secured than those with part-time jobs. According to Bonti-Ankomah (2001), the main challenge of food security in South Africa is access to food. This is because food access is determined by demand and purchasing power. Another problem exacerbating food insecurity in South Africa is high unemployment, which limits many households in purchasing food. Figure 3 demonstrates trends of food adequacy at household and provincial level, respectively.

The survey shows that over 80% of people in eight provinces have adequate food access, except in Free State (FS), averaging below around 70%. The survey further indicates that during 2015, food inaccessibility was most serious in Free State where 20% of the households have inadequate food access, followed by KwaZulu-Natal (KZN) at 15% and Eastern Cape at around 10%. Western Cape (WC) had the least food security problems at around 5%, followed by Limpopo (LP) at around 4.3%. According to the FAO 2008 report, high levels of unemployment and inadequate social welfare are the major contributors to food insecurity in South Africa.

The South African government uses various methods to assess food security. These include General Household Survey (GHS), Income and Expenditure Index (IES), Labour Force Survey (LFS) and Community Survey (Statistics South Africa 2015). However, due to the complexity of measuring food security, these methods yield different results. The FAO (2008) reports that black South Africans constitute the majority of poor and food insecure households, which are largely found in rural areas. Factors such as lack of access to finance, communication infrastructure, education and skill development facilities prevent households from engaging in agricultural farming. In 2008, the World Bank (2010) estimated that 39.26% of the total South African population live in rural areas and 65% of them were identified as poor. These statistics suggest that interventions to combat food insecurity in South Africa should largely be directed to rural areas. This is further emphasised by Machete (2004) arguing that the South African government needs to give more support to the small-scale farmers to improve agricultural production, particularly at the rural household level.

In summary, countries differ in terms of the exposure and vulnerability to climate change. Therefore, South Africa’s ability to adapt and protect its food security will depend on the understanding of risks and the vulnerability of various food items to changes in climate. More importantly, understanding how such spillover effects will affect households, particularly in rural areas as they rely heavily on agriculture, is important given that South Africa is currently faced with droughts, resulting in lack of rainfall, rivers drying out, loss of livestock and farmers’ production decreasing very significantly. From Table 2, it can be noted that household is a significant determinant for food security. It is therefore important for the government to implement strategies to address food security at the household level. Furthermore, it is vital to educate communities, particularly in rural areas, on how to maintain their farming during harsh climate changes. The section below outlines efforts taken by the government to assist household farming.

**TABLE 2:** The Impact of climate change on food security.

Climate change	Impacts
Increase in average temperature	Reduced quantity and reliability of agricultural yield
	Increased heat stress in livestock
	Destruction of crops or lowering crop productivity
	Decline in certain fish stocks due to increased sea temperature
Change in amount of rainfall	Reduced water availability for crop and livestock
	Heavy reliance on irrigation
	Poor quality of crops due to deteriorating water quality
Increased severity of drought	Decreased crop yield
	Increased probability of fire
Increased intensity of extreme events	Soil erosion
	Increased land degradation and desertification
	Inability to cultivate land
	Damage to crops and food stores

*Source*: Compiled by the author, adopted from the World Wide Fund (WWF), 2014, Understanding the food energy nexus: Climate change, the food energy water nexus and food security in South Africa, British High Commission, Pretoria

## The role of Department of Agriculture, Forestry & Fisheries

South Africa’s integrated food security strategy of 2002 identified specific challenges for food security. Some of the key areas include:

- weak institutional support networks and disaster management systems
- inadequate and unstable household food production
- lack of purchasing power
- poor nutritional status.

The strategic document also specified priority areas to address challenges of food security, namely:

- increasing household food production
- increasing food trade and distribution
- increasing income opportunities
- improving nutritional status
- enhancing institutional support networks
- providing capacity building.

The integrated food security strategy serves as a guideline to the DAFF to contribute towards addressing food security in South Africa. In addition, the DAFF developed a Zero Hunger Strategy, which is aimed at combating hunger. The objectives of the strategy include:

- to provide an effective mechanism for the coordination of national, provincial and Non-Governmental Organisations (NGOs) to pursue a common goal of increasing food security
- to ensure the establishment of effective support structures for farmers through capacity building and institutional strengthening
- to demonstrate opportunities for diversification and increasing income through the production of vegetables, small stock and small-scale aquaculture
- to build an effective capacity at the local level through intensive training and access to information that will provide support to the farming communities
- to evaluate the impact of the interventions, to identify gaps and quantify constraints that still need to be addressed and to make recommendations.

Another programme developed by the DAFF is the Production Strategy. The strategy aims to position primary agriculture by targeting subsistence farmers, small-scale farmers and commercial production for the purpose of improving food safety and security in South Africa. The strategy identifies five pillars of food security: food availability, affordability, stability of supply, food accessibility and utilisation. It is the responsibility of the provincial and local governments to ensure that the national programmes to address food security reach the communities (Department of Environmental Affairs 2013).

## Conclusion and recommendations

The purpose of this article was to examine the impact of climate change on food security in South Africa. This was accentuated by the occurrence of drought that affected the country recently in 2016. From the discussion, food security was found to be a multifaceted concept affected by climate change through food accessibility, availability, accessibility, utilisation and affordability. Agriculture plays a vital role in ensuring food security; it is therefore important to eradicate greenhouse emissions. Figure 2 demonstrates that food insecurity is further exacerbated by an increase in population which is greater than the rate of food production, resulting in food shortage. With the lack of rainfall and drought, South Africa is likely to face food insecurity in the future. Another concern is the country’s high levels of unemployment and poverty, which limit many households to access and purchase food. This can further worsen risks of food insecurity. This article therefore suggests that a fight against food insecurity requires not a blanket approach but a multipronged strategy to address other socio-economic challenges such as poverty and unemployment. The article further established that the vulnerability of food security to climate change differs from country to country. Therefore, South Africa’s ability to adapt and protect its food security will depend on the understanding of risks and the vulnerability of various food items to changes in climate. This is important given that South Africa is currently faced with droughts, resulting in the lack of rainfall, rivers drying out, loss of livestock and farmer’s production decreasing very significantly. This article shows how arable land fit for agriculture is becoming scarce through global warming. This calls for a technological intervention, so that agriculture food items can resist the effects of global warming. However, the challenges of adapting to climate changes in most developing countries, including South Africa, are more difficult because such countries have weak institutions and limited access to technology. Another concern is a wide gap between the cost of adapting to climate change and the necessary financial and educational support from the government. There is also a need to invest in technologies that will resist and manage climate risks to food security and also to address the vulnerabilities and weaknesses in food systems.
